# Retinoblastoma Cell Growth *In Vitro* and Tumor Formation *In Ovo*—Influence of Different Culture Conditions

**DOI:** 10.3390/mps5020021

**Published:** 2022-03-02

**Authors:** Annika Doege, Rebecca Steens, Nicole Dünker, Maike Anna Busch

**Affiliations:** Center for Translational Neuro- and Behavioral Sciences (C-TNBS), Institute of Anatomy II, Department of Neuroanatomy, Medical Faculty, University of Duisburg-Essen, 45147 Essen, Germany; annika.hubert@stud.uni-due.de (A.D.); rebecca.steens@stud.uni-due.de (R.S.); nicole.duenker@uk-essen.de (N.D.)

**Keywords:** retinoblastoma, CAM assay, chorioallantoic membrane, cell culture, EMT

## Abstract

Retinoblastoma (RB) is a primary intraocular malignancy in childhood. Relapses may develop and cause secondary cancers during later development. This study was set up to identify optimal cell culture conditions for RB cell growth *in vitro* and to optimize tumor growth in an *in vivo* model. RB cell lines (Y79 and WERI-Rb1) were cultivated under three different *in vitro* conditions and apoptosis, proliferation and cell growth, as well as expression profiles of two epithelial-mesenchymal transition (EMT) markers, were analyzed. EMT gene expression profiles were not generally changed, whereas apoptosis levels, tumor cell proliferation, and *in vitro* growth were significantly influenced by different cell culture conditions. In order to optimize the time-limited chick chorioallantoic membrane (CAM) assay, we investigated two different time points of tumor cell inoculation (embryonic development day EDD8 and EDD10) as well as three different cell concentrations. We showed that inoculation at EDD8 led to decreased tumor formation and chicken viability, whereas different cell concentrations did not change size and weight of developing tumors. Our findings demonstrate that medium conditions *in vitro* as well as the starting point for CAM inoculation *in ovo* significantly influence the experimental outcome of investigations using RB cell lines.

## 1. Introduction

Retinoblastoma (RB) is the most common malignant pediatric intraocular tumor [1], representing 2.5–4% of all pediatric cancers, and approximately 9000 new cases develop each year worldwide [1,2]. The total survival rate is high with a good eye-salvage rate (95%); saving vision, however, fails in advanced cases [3]. Untreated tumors expand and may extend beyond the eye causing metastatic spread [2]. Thirty-five percent of RB patients develop secondary tumors and 50% of these patients die after therapy [4].Therefore, reliable *in vitro* and *in vivo* systems are indispensable for studying RB tumor biology in order to develop new treatment strategies for an optimization of RB management. However, different cultivation protocols for the same RB cell lines have been published so far [3,5,6,7,8]. It is well-known that varying cell culture conditions, e.g., using different media and/or supplements as well as different CO_2_ concentrations, have a huge impact on the tumor cell growth, viability and gene expression, e.g., of epithelial-mesenchymal transition (EMT) markers. Therefore, it is comprehensible that experiments using the same RB cell lines are still not verifiable across different labs as they are carried out using different cultivation protocols.

The chick chorioallantoic membrane (CAM) assay is an established model system for *in vivo* studies of tumor cell growth and dissemination [9,10] as well as for drug testing [11,12]. The CAM forms within 4 to 5 days of avian embryonic development by the fusion of the mesodermal layers of the chorion and allantois. The chick embryo itself hatches at embryonic development day (EDD) 21 [10,11]. The highly vascularized CAM stimulates the growth of grafted tumor cells and the model itself is naturally immunodeficient, allowing for the inoculation of tumor cells and tissues without species-specific restrictions [11]. The extraembryonic vessel system is easily accessible for manipulation and observation of tumor formation and angiogenesis [11,13]. Previous publications reported on experimental setups to obtain tumor growth on the CAM, starting inoculation at different EDDs and grafting various tumor cell lines and primary tissues [13,14,15]. 

Our group successfully established the CAM assay for the analysis of RB tumor growth and metastatic spread [6,16,17]. However, different protocols have been described for this method when it comes to cell concentrations being used and given time points for starting the inoculation of cells, ranging from EDD3 up to EDD3 [17,18,19,20,21]. A standard operating procedure (SOP) for CAM assays using RB cell lines is still missing.

Thus, in the study presented, we systematically investigated differences in growth behavior and gene expression of two RB cell lines (Y79, WERI-Rb1) grown under three commonly used *in vitro* culture conditions. Additionally, we re-investigated our established *in ovo* CAM protocol with three RB cell lines (Y79, WERI-Rb1 and RB355) to compare different conditions for *in ovo* tumor growth of RB cells and to identify the most suitable experimental setup.

## 2. Material and Methods

### 2.1. Cell Culture

The human RB cell lines Y79 [5] and WERI-Rb1 [22] were originally purchased from the Leibniz Institute DSMZ (German Collection of Microorganisms and Cell Cultures). The RB cell line RB355 was established and first described by Griegel et al. [3]. All RB cell lines were kindly provided by Dr. H. Stephan. Sequencing of the *RB1* gene was performed for all retinoblastoma cells lines.

Two cell lines (Y79 and WERI-Rb1) were cultivated at 37 °C and 95% humidity using the following three different media and CO_2_ conditions:

(1) RB medium composed of Dulbecco’s modified Eagle’s medium (DMEM; PAN-Biotech; Aidenbach; Germany; Cat. No.: P04-04510) with 15% fetal bovine serum (FBS; PAN-Biotech; Aidenbach; Germany; P30-3702), 100 U penicillin/ml and 100 µg streptomycin/ml (Gibco; Grand Island, NEB, USA; Cat. No.: 15140), 4 mM L-glutamine (Gibco; Grand Island; USA; Cat. No.: 25030-024), 50 µM ß-mercaptoethanol (Roth; Germany; Karlsruhe; Cat. No.: M6250) and 10 µg insulin/ml (PAN-Biotech; Aidenbach; Germany; Cat. No.: P07-04300). Cultivation at 10% CO_2_.
(2) DMEM medium composed of Dulbecco’s modified Eagle’s medium (DMEM; PAN-Biotech; Aidenbach; Germany; P04-04510) with 15% fetal bovine serum (FBS; PAN-Biotech; Aidenbach, Germany; P30-3702), 100 U penicillin/ml and 100 µg streptomycin/ml (Gibco; Grand Island; USA; Cat. No.: 15140) and 4 mM L-glutamine (Gibco; Grand Island; USA; Cat. No.: 25030-024). Cultivation at 5% CO_2_.
(3) RPMI medium containing biotin, vitamin B_12_ and PABA (Gibco; Grand Island; USA; Cat. No.: 11875-093) with 15% fetal bovine serum (FBS; PAN-Biotech; Aidenbach, Germany; P30-3702), 100 U penicillin/ml and 100 µg streptomycin/ml (Gibco; Grand Island; USA; Cat. No.: 15140) and 4 mM L-glutamine (Gibco; Grand Island; USA; Cat. No.: 25030-024). Cultivation at 5% CO_2_.

The RB cells used for the *in ovo* CAM assays (Y79, WERI-Rb1 and RB355) were cultivated in RB medium as described above. No approval from an ethics committee was required for work with the human cell lines.

### 2.2. Cell Proliferation and Apoptosis Detection

Cell proliferation was investigated by 5-Bromo-2′-deoxyuridine (BrdU; Sigma; Darmstadt, Germany; Cat. No.: S B9285) incorporation. Therefore, 10 µM BrdU was added to the cells 4 h prior to paraformaldehyde (PFA; Sigma; Darmstadt, Germany; Cat. No.: P6148-500G) fixation procedure. Afterwards, cells were incubated with a rat anti-BrdU antibody (1:1000; ab6326; Abcam; UK) and proliferating cells were finally detected using a goat anti-rat antibody labeled with Alexa Flour**^®^** 488 (1:1000; A11006; Life Technologies; Germany). In order to detect changes in apoptosis levels, cells were stained with 4′,6-Diamidino-2-phenylindole (DAPI; Sigma; Darmstadt, Germany; Cat. No.: D9542) and pycnotic nuclei were counted as described previously by our group [23].

### 2.3. Growth Curves 

To determine growth kinetics, 3 × 10^5^ RB cells were seeded in 500 µl of each medium (RB medium, DMEM medium and RPMI medium) with supplements in a 24-well plate and vital cells were counted manually using the trypan blue exclusion method. Cells were seeded in triplicates and counted at four time points (24 h, 48 h, 72 h, 96 h).

### 2.4. Gene Expression Analysis

RNA isolation from RB cell lines was performed using the NucleoSpin RNA II kit (Macherey and Nagel; Düren, Germany; Cat. No.: 740955.250). For quantitative real-time PCR analyses, cDNA was synthesized with the QuantiTect Reverse Transcription Kit (Qiagen, Hilden, Germany; Cat. No.: 205313) following the manufacturer’s protocol. For analysis of *vimentin* and *EpCAM* expression, a SYBR™ Green PCR assay (Applied Biosystems; Waltham, MA, USA) was used with specific primers 5′-TCTGGATTCACTCCCTCTGGT-3′ (forward) and 5′-TCAAGGTCA TCGTGATGCTGA-3′ (reverse) for whole *vimentin*, 5′-TCAGAATGAT GTGGACATAGCTGA-3′ (forward) and 5′-CCCCATTTACTGTCAGGTCCA-3′ (reverse) for whole *EpCAM* and 5′-ACCCACTCCTCCACCTTTGA-3′ (forward) and 5′-CTGTTGCTGTAGCCAAATTCGT-3′ (reverse) for human *GAPDH* (hGAPDH) as an endogenous control. RT-PCRs were conducted in triplicates in 20 μL of SYBRTM Green PCR Master Mix (Applied Biosystems, Waltham, Massachusetts, USA; Cat. No.: 4309155) using the following program: 95 °C for 15 min; 94 °C for 15 s, 55 °C for 30 s, and 70 °C for 34 s and 40 cycles.

### 2.5. CAM Assays

In order to test for changes in the tumor formation capacity and mortality rate of the chicken embryos, different RB cell concentrations (1 × 10^6^, 2 × 10^6^ and 3 × 10^6^ cells) were inoculated onto the chick chorioallantoic membrane (CAM) at embryonic development day (EDD) 8 and EDD10 eggs as described below and previously published by our group [6]. Mainly following published CAM metastasis model protocols [24,25], 50 μl cell suspension were inoculated onto the exposed CAM area. For each RB cell line (Y79, WERI-Rb1 and RB355) at least 10 eggs were grafted in at least 3 independent experiments, respectively.

### 2.6. Grafting of RB Cells

Fertilized eggs were incubated in a humidified rotary incubator at 38 °C and 50% humidity for 8 or 10 days. Thereafter, the eggs were candled by shining light into the eggshell in order to identify the chorioallantoic vein. Then a 1 cm square was marked approximately 1 cm away from the veins branching point. A hole was drilled through the blunt end of the egg into the air sac and the previously drawn square in order to drop the CAM by gentle suction, creating an artificial air sac. The CAM was gently abraded by the use of a cotton-tipped applicator. Thereafter, the cell suspension (in PBS) was placed onto the traumatized CAM area. The window in the eggshell was sealed tightly with tape and the egg was returned to the incubator until EDD17. 

### 2.7. Harvesting of Tissue

According to the German Animal Experiment and Welfare Guidelines, no ethics approval is required for *in ovo* experiments if the chicken is not intended to live beyond hatching. In our study CAM assays were only performed until day EDD17 and thus, prior to hatching. Thus, the duration of the CAM assay was limited to a 7–9 day time window. Seven to nine days after grafting (EDD10-17 or EDD8-17), chick embryos were anesthetized by cooling on ice and sacrificed by decapitation. Mortality rate of the chicken embryos were determined and CAM tumors were excised, measured, and photographed. 

### 2.8. Statistical Analysis

All assays were performed at least in triplicate and data represent means ± SEM from independent RB cell cultures. Statistical analyses were conducted using GraphPad Prism 6. Data were analyzed by Student’s *t*-test or one-way ANOVA and Newman-Keuls Post-test. * *p* < 0.05, ** *p* < 0.01 or *** *p* < 0.001 were considered significantly different. For the statistics on the growth curves a free web interface http://bioinf.wehi.edu.au/software/compareCurves/ was used (Accessed on 1 December 2021), which uses the compareGrowthCurves-function from a statistical modeling package called ‘statmod’, available from the R Project for Statistical Computing: http://www.r-project.org (accessed on 1 December 2021), previously described elsewhere [26].

## 3. Results

### 3.1. RB Cell Line Morphology under Different Cell Culture Conditions

**Figure 1:**
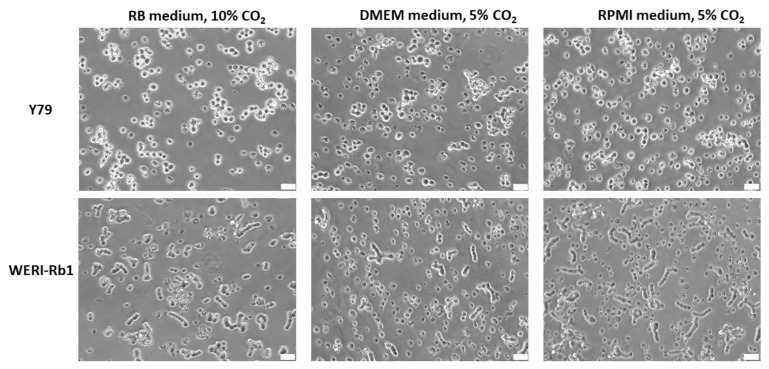
Morphology of Y79 and WERI-Rb1 RB cells in suspension culture grown under the different culture conditions indicated. Phase contrast microscopy photos, white scale bar: 32 µm.

The established human RB cell lines Y79 and WERI-Rb1 grew in suspension under all conditions analyzed and appeared as small epithelioid round cells (Figure 1), mainly forming cell clusters and aggregates. WERI-Rb1 cells frequently tend to build cell chains which, however, did not resemble Flexner-Wintersteiner rosettes (Figure 1). No difference in cell morphology was discernable between the three different cell culture conditions tested.

### 3.2. Different Culture Conditions Changed Apoptosis, Proliferation and Growth Kinetics of RB Cell Lines

**Figure 2:**
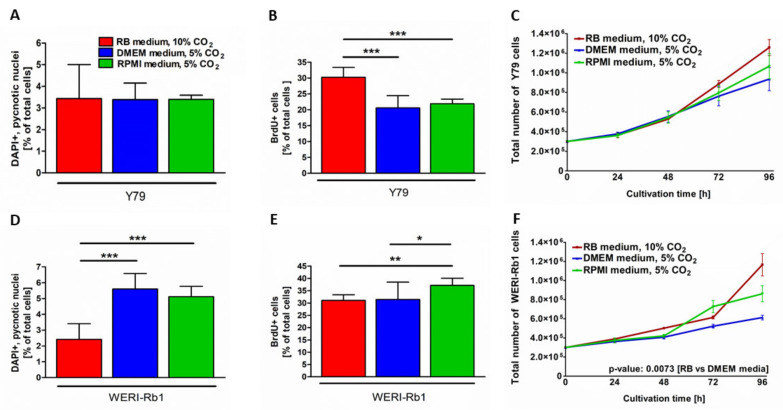
Effects of different cell culture conditions on apoptosis, proliferation, and growth kinetics of Y79 and WERI-Rb1 RB cell lines. The different cell culture conditions had an impact on apoptosis, proliferation and RB cell growth of Y79 and WERI-Rb1 cells as revealed by DAPI cell counts (**A**,**D**), BrdU stains (**B**,**E**) and growth curves (**C**,**F**). Values are means of three independent experiments ± SEM. * *p* < 0.05, ** *p* < 0.01, and *** *p* < 0.001; statistical differences calculated by one-way ANOVA with Newman–Keuls post-test.

Depending on the RB cell line, significant changes in apoptosis and/or proliferation rates as well as differences in cell growth were observed under the three different growth conditions investigated. While culture conditions seemed to have no influence on the endogenous apoptosis rate of Y79 cells (Figure 2A), cell death levels were significantly increased in WERI-Rb1 cells cultivated in DMEM and RPMI media and 5% CO_2_ compared to cells grown in RB medium and 10% CO_2_ (Figure 2D). By contrast, DMEM and RPMI media conditions significantly decreased the proliferation rate of Y79 cells compared to RB medium conditions (Figure 2B), whereas RPMI medium conditions seemed to have a positive effect on WERI-Rb1 proliferation compared to RB and DMEM media conditions (Figure 2E). For both cell lines growth kinetics showed the highest overall cell growth in RB medium under 10% CO_2_ and the lowest in DMEM medium under 5% CO_2_ (Figure 2C,D), however, only reaching significance for WERI-Rb1 cells grown under RB medium conditions compared to DMEM medium conditions (Figure 2F). Taken together RB cells grown under RB medium conditions displayed the lowest overall apoptosis and highest proliferation rate, resulting in higher growth kinetics in comparison to the other conditions investigated.

### 3.3. Vimentin and EpCAM Gene Expression Analysis under Different Cell Culture Conditions

**Figure 3:**
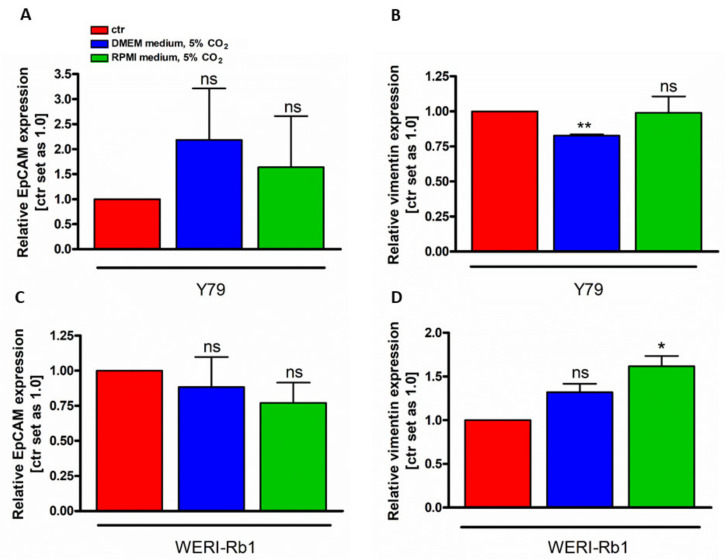
*EpCAM* (**A**,**C**) and *vimentin* (**B**,**D**) gene expression analysis of Y79 and WERI-Rb1 cells under different cell culture conditions. Values are means of three independent experiments ± SEM. ns: not significant, * *p* < 0.05, ** *p* < 0.01, statistical differences calculated by Student’s *t*-test. ctr: RB medium, 10% CO_2_.

In order to investigate if the cell identity changed in RB cell lines grown under different culture conditions, the gene expression status of two EMT markers, the mesenchymal marker *vimentin* and the epithelial marker *EpCAM* were performed. We compared DMEM and RPMI media conditions with RB medium condition. No significant *EpCAM* gene expression changes were detectable (Figure 3A,D). *Vimentin*, however, showed cell line and cell culture condition dependent gene expression changes, with decreased expression in Y79 cells grown under DMEM medium conditions (Figure 3A) and increased expression of WERI-RB1 cell cultured under RPMI medium conditions. 

### 3.4. Systematic Analysis of In Ovo RB Tumor Growth in the CAM Model

**Figure 4:**
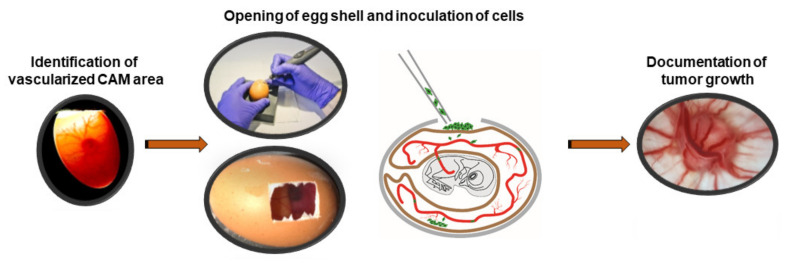
*In ovo* CAM model for tumor growth after inoculation of RB tumor cells.

To address the question, which experimental conditions are favorable for RB tumor growth *in ovo*, we compared two different CAM inoculation time points as well as three different cell concentrations. For this purpose, at embryonic development day (EDD) 8 and EDD10, a highly vascularized CAM area was identified by candling the eggs, marked and the eggshell was opened. After inoculating different RB cell concentrations (1, 2, or 3 × 10^6^ cells) onto the dropped CAM, eggs were re-incubated until EDD17 (Figure 4). Subsequently, we investigated the mortality rate of the chicken embryos and harvested the developed tumors from three different RB cell lines investigated (WERI-Rb1, Y79 and RB355) in order to measure the size and weight. 

### 3.5. In Ovo RB Cell Tumor Development and Mortality Rate of Chick Embryos Depend on the Time Point of CAM Experiments

**Figure 5:**
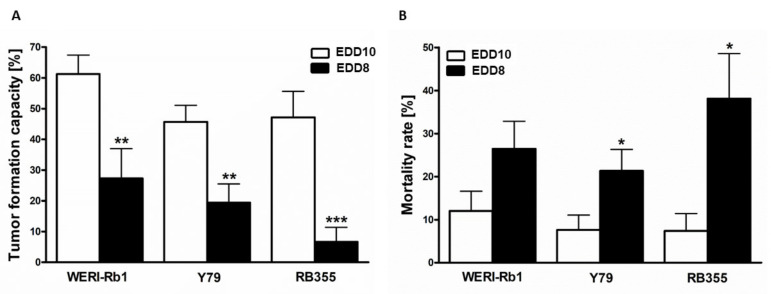
Effects of different inoculation time points on tumor formation capacity and chicken mortality in ovo. Tumor formation capacity of all three RB cell lines (WERI-Rb1, Y79 and RB355) is significantly decreased upon inoculation at embryonic development day eight (EDD8) in comparison to embryonic development day ten (EDD10) (**A**). The mortality rate of the chicken embryos was increased for all RB cell lines after inoculation on EDD8 in comparison to EDD10 (**B**). Values are means of three independent experiments ± SEM. * *p* < 0.05, ** *p* < 0.01, *** *p* < 0.001; statistical differences calculated by Student’s *t*-test.

To investigate whether the time point of CAM inoculation influences the general tumor formation capacity of RB cells and mortality of the chicken embryos, we inoculated three different RB cells lines onto the CAM, once on EDD8 and once on EDD10. At EDD17, all RB cell lines investigated displayed significantly decreased tumor formation following inoculation at EDD8 compared to inoculation at EDD10 (Figure 5A). In addition, the mortality rate of the chick embryos was significantly increased after inoculation of Y79 and RB355 cells at EDD8 (Figure 5B). For WERI-Rb1 cells this effect did not reach significance. 

### 3.6. Inoculated RB Tumor Cell Concentration Did Not Influence Tumor Size and Weight In Ovo

**Figure 6:**
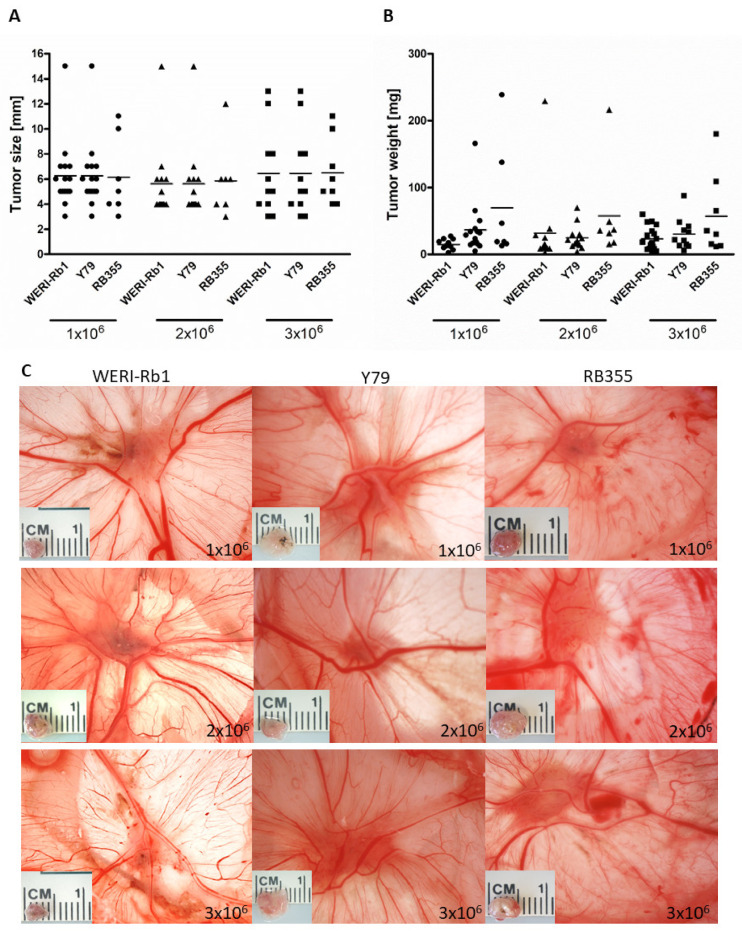
Effects of different RB cell concentrations on tumor size and weight *in ovo*. Tumor size (**A**,**C**) and weight (**B**) of all three RB cell lines (WERI-Rb1, Y79 and RB355) are not significantly influenced by different cell concentrations (1, 2 or 3 × 10^6^ cells) inoculated on embryonic day (EDD10). Values are means of three independent experiments ± SEM. The symbols in (**A**) and (**B**) represent individual eggs used for grafting the following RB cell concentrations: circles: 1 × 10^6^ cells, triangles: 2 × 10^6^ cells, squares: 3 × 10^6^ cells.

As we could show that inoculating RB tumor cells at EDD10 leads to higher chicken viability and significantly higher tumor formation, we analyzed the impact of different cell concentrations inoculated at EDD10. We could show that inoculation of different RB cell concentrations (1–3 × 10^6^ cells) did not change the tumor size (Figure 6A,C) and tumor weight (Figure 6B) of any RB cell line investigated. Inoculation at EDD8 likewise revealed no differences in tumor size and weight (data not shown). With regard to often limited cell numbers in experimental settings, 1 × 10^6^ RB tumor cells are suitable and sufficient for inoculation at EDD10 and lead to an average RB tumor size of 6 mm and tumor weight of 34 mg. 

## 4. Discussion

Although it is commonly accepted that the tumor microenvironment plays a crucial role for tumor behavior [27], 2D cell cultures are still an important component of tumor investigations as they are easily accessible, give insights into tumor biology and gene expression patterns, and are indispensable for new drug development studies. Over the past decades, several cultivation protocols for RB cells have been routinely used in different experimental settings. However, so far, little attention has been paid to *in vitro* culture conditions used to grow RB tumor cells, notwithstanding that medium conditions like serum and growth factors, glucose and CO_2_ concentrations influence tumor cell growth as well as gene expression of tumor cells [28]. 

We therefore compared three different cultivation settings commonly used for RB cell cultures [3,5,6,7,8] in order to identify differences in morphology, growth and gene expression patterns. We could show that RB morphology is not affected by the culture conditions analyzed. Nevertheless, we identified significant changes in apoptosis, proliferation rates and cell growth. The effects are partially cell line dependent, but taken together, growth in RB medium condition resulted in lowest apoptosis rates and highest RB cell growth, which reflects the most favored properties of tumor cell cultures. Additionally, we analyzed gene expression patterns of the two EMT markers *vimentin* (mesenchymal marker) and *EpCAM* (epithelial marker) to detect changes in cellular fate upon different medium conditions. We could show that with regard to potential EMT changes the identity of the RB tumor cells did not significantly alter or differ between the medium conditions except for *vimentin* expression being upregulated in WERI-Rb1 cells cultivated in RPMI medium and downregulated in Y79 cultured in DMEM medium compared to RB medium conditions. Taken together the results of the EMT marker expression analyses support our observations that the morphology of the RB cells did not change as no shift towards a mesenchymal or epithelial cell morphology was discernible.

Beside *in vitro* cell cultures, *in vivo* experiments are inevitable to investigate tumor formation in a more natural environment. One *in vivo* model system, which offers a plethora of advantages over the standard murine model, is the *in ovo* chicken chorioallantoic membrane assay. The CAM is at least partially immune-deficient and thus, allows for growth analyses of different tumor entities and species without specific or nonspecific immune response. Additionally, the rich blood vessel network of the CAM promotes survival, growth, and rapid vascularization of CAM tumors formed by inoculated cancer cell, xenografted normal or tumor tissues (for review see: [10,12,17,21]). Against the background that the duration of the CAM assay is limited to a time window well before chick embryo hatching, we intended to investigate if an earlier inoculation time point leads to increased tumor formation. Therefore, we compared tumor cell inoculation at embryonic development day (EDD) 10 with an earlier time point (EDD8) and additionally tested different cell concentrations with regard to tumor formation capacity and chicken mortality. We could clearly show that the earlier time point of inoculation (EDD8) is not advantageous, even if the tumor cells have two more days to grow compared to inoculation at EDD10, when terminating the experiments at EDD17. Quite contrary to the expected benefit for tumor growth, the RB tumor formation capacity was significantly reduced for all three RB cell lines tested after inoculation at EDD8. In addition, the mortality rate of the chicken embryos is increased, reaching significance for Y79 and RB355 RB cell lines. Different cell concentrations inoculated did not change the tumor formation capacity, tumor size and weight. Thus, a fully developed capillary plexus connecting the arterial and venous blood vessels at EDD10 [17] seems to play a pivotal role for tumor development on the CAM and even chicken viability throughout the experimental time period. 

In summary, we could show that medium conditions *in vitro* as well as time point of CAM inoculation *in ovo* significantly influence the experimental outcome of investigations using RB cell lines. RB medium combined with a 10% CO_2_ atmosphere turned out to be the most suitable *in vitro* culture condition for RB cell lines. EDD10 is the preferential starting point for CAM inoculation assays due to better overall embryo survival and tumor development. It seems that the cell concentration used for inoculation experiments does not significantly influence *in ovo* tumor development of RB cells. Taken together, it would be desirable to optimize and standardize experimental *in vitro* and *in ovo* conditions in order to gain reproducible research results. 

## Data Availability

Not applicable.
